# Addressing the Limits of Emx2 Therapy of Glioblastoma Multiforme by Transgene Insulation and Epigenetic Pharmacological Intervention

**DOI:** 10.3390/biomedicines14092010

**Published:** 2026-09-07

**Authors:** Mariacarmine Tuccillo, Olga Pastorino, Carmen Falcone, Jessica Zucco, Giampiero Leanza, Antonello Mallamaci

**Affiliations:** 1 Department of Neuroscience, Scuola Internazionale Superiore di Studi Avanzati (SISSA), Via Bonomea 265, 34136 Trieste, Italy; 2 Present Affiliation: Telethon Institute of Genetics and Medicine (TIGEM), 80078 Pozzuoli, Italy; m.tuccillo@tigem.it; 3 Present Affiliation: Independent Researcher, 34136 Trieste, Italy; olga.pastorino@gmail.com; 4 Present Affiliation: Department of Biological Sciences, Towson University, 8000 York Road, Towson, MD 21252, USA; cfalcone@towson.edu; 5 Present Affiliation: Institute of Medical Genetics, University Hospital ‘Santa Maria della Misericordia’, Azienda Sanitaria Universitaria Friuli Centrale (ASUFC), Piazzale Santa Maria della Misericordia, 15, 33100 Udine, Italy; jessica.zucco@asufc.sanita.fvg.it; 6 Department of Life Sciences, University of Trieste, Via Fleming 22, 34127 Trieste, Italy; 7 Present Affiliation: Department of Drug and Health Sciences, University of Catania, Viale A. Doria 6 (Bldg 2), 95125 Catania, Italy; giampiero.leanza@unict.it

**Keywords:** Emx2, glioblastoma, gene therapy, gene silencing, gene insulation

## Abstract

**Introduction/Objectives:** Implicated in the regionalization of the anterior central nervous system (CNS) and the progression of pallial astrogliogenesis, the transcription factor gene *EMX2* has been reported to be specifically silenced in several tumors, both non-neural and neural. Based on that, its overexpression has been proposed as a tool for the treatment of a subset of these malignancies, including glioblastoma. Here we tested this proposal. **Methods/Results:** We found that *Emx2* overexpression in human glioblastoma cells transplanted into the striatum of immunotolerant mice significantly prolonged animals’ survival, outperforming their treatment by temozolomide. However, this approach did not eradicate the tumor, because of in vivo silencing of the therapeutic *Emx2* transgene. Notably, silencing of this transgene (or of a reporter designed to monitor its competence to be expressed) also occurred during long-term in vitro culture of GBM cells and was exacerbated by coculture with murine pallial glia under hypoxic conditions. Remarkably, partial insulation of the transgene and the use of a specific combination of epigenetic drugs substantially counteracted the progressive activatability decline undergone by the *Emx2*-transgene expression reporter, suggesting a promising strategy for overcoming a major limitation of *Emx2*-based therapy for GBM. **Conclusions:** An Emx2-encoding transgene, protected by insulation and appropriate epigenetic drugs, can provide a substantial benefit in experimental therapy for glioblastoma.

## 1. Introduction

Implicated in early regionalizazion of the anterior CNS along rostro-caudal and dorso-ventral axes [1,2,3], the transcription factor Emx2 subsequently commits pallial stem cells to astroglial fates, through the upregulation of *Couptf1*, *Nfia* and *Sox9* [4], and finally drives committed glial progenitors to exit cell cycle and differentiate into astrocytes [4], by downregulating *Fgf9* and *EgfR* [5]. Consistently with such anti-proliferative activity, an inverse correlation between Emx2 expression levels and tumor aggressiveness has been reported in a wide range of malignancies, including human gastric cancer [6], endometrial cancer [7], renal cell carcinoma [8,9], malignant pleural mesothelioma [10], lung squamous cell carcinoma [11], colorectal cancer [12,13], esophageal adenocarcinoma [14], sarcoma [15,16], and glioblastoma [17,18,19]. Moreover, the epigenetic silencing of *EMX2* following a reconfiguration of the EZH2-dependent H3K27 methylation landscape was proposed to pave the way to glioma progression to high grade malignancy [18]. Consistently, reactivation or overexpression of *Emx2*/*EMX2* in GBM cells was shown to exert a powerful antitumor activity, in vitro as well as in vivo [17,18,19]. This activity has been demonstrated across multiple GBM models, including both established cell lines and primary patient-derived cultures. It is mediated by EMX2-dependent modulation of a variety of key effectors critical for tumor maintenance and malignancy [17,18,19]. This includes functionally relevant, *EGFR* and *SOX2* downregulation, *HES1* upregulation, Erk1/2 signaling suppression, pSmad1/5/8 signaling stimulation, and EIF4E translation factor inhibition [17]. These findings identify Emx2/EMX2 as a promising therapeutic candidate for the treatment of diverse GBM subtypes, including recurrent tumors.

Here, we performed a proof-of-concept study to assess the feasibility of this therapeutic strategy, by overexpressing *Emx2* in U87MG cells transplanted into the striatum of adult immune-compromised mice. Despite the remarkable increase in the survival time of these animals, the long-term therapeutic efficacy of *Emx2* gene transfer was limited by progressive transgene silencing. We further examined the contribution of glial cells and hypoxia to this phenomenon and assessed whether transgene hemi-insulation combined with epigenetic drug treatment could preserve transgene expression and thereby improve the durability of the therapeutic response.

## 2. Materials and Methods

### 2.1. Lentiviruses

Recombinant lentiviruses were generated and titrated as described [20]. For this purpose, the following cargo plasmids were employed:

- LV_Pgk1p-tTA-wpre
- LV_Pgk1p-rtTA^M2^-wpre
- LV_TREt-Ires-Egfp-wpre
- LV_TREt-Emx2-Ires-Egfp-wpre
- LV_TREt-E2L-Ires-Egfp-wpre
- LV_Ins-TREt-Ires-Egfp-wpre
- LV_Ins-TREt-E2L-Ires-Egfp-wpre
- LV_Pgk1p-mCherry-wpre

**Figure 1:**
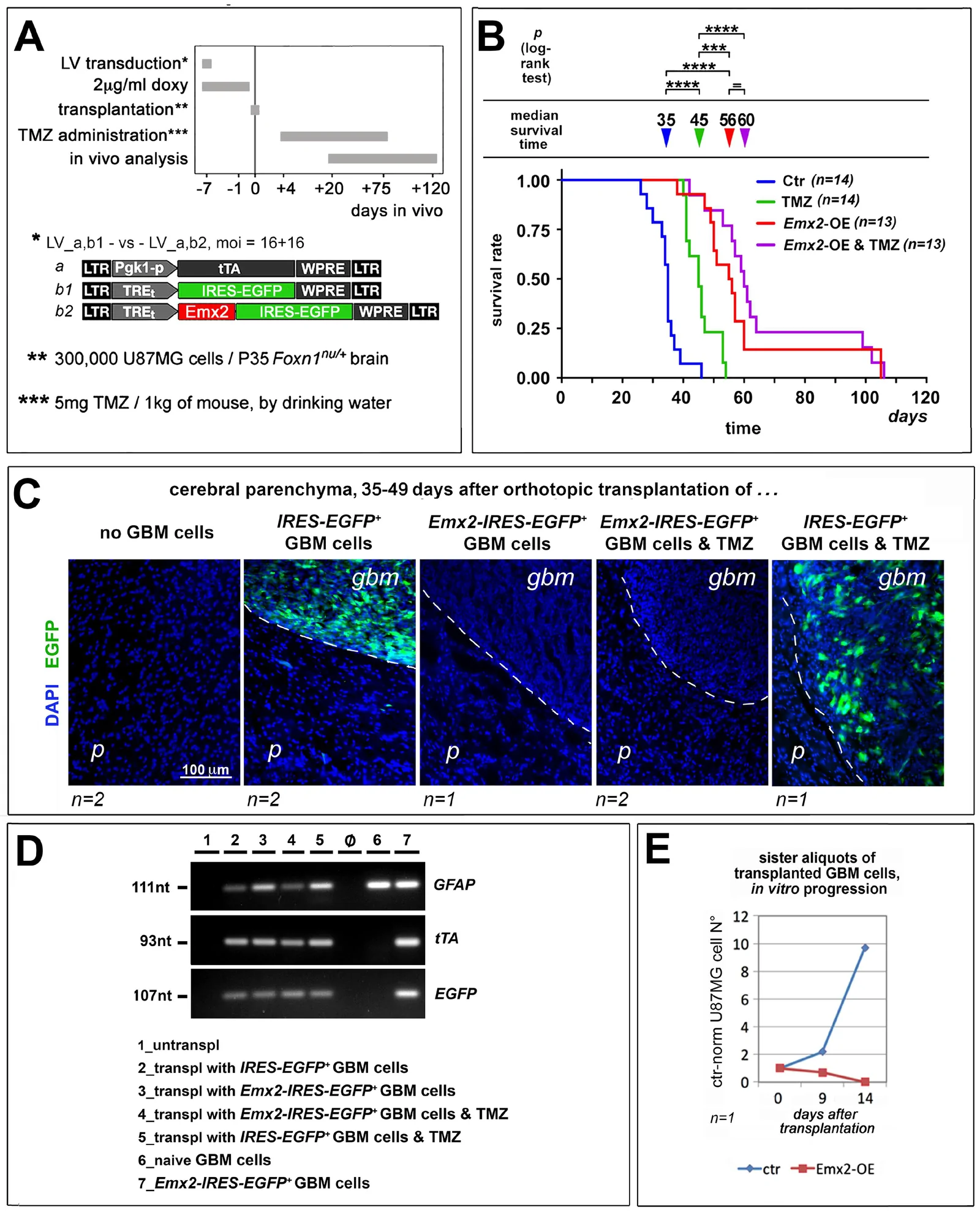
Emx2-based anti-GBM therapy in immunocompromised mice xenotransplanted with engineered U87MG cells. (**A**) Experimental protocol. It includes temporal articulation of the procedure, lentiviruses employed for GBM cell engineering, key transplantation details and TMZ doses administered. (**B**) Survival of xenotransplanted mice. Kaplan-Meier curves, with median survival times. *n* is the number of transplanted animals included into the four experimental groups. Statistical significance of differences evaluated by log-rank test. =, not significant; ***, *p* < 10^−3^; ****, *p* < 10^−4^. (**C**) Distribution of anti-EGFP immunoreactive cells in transplanted brains. Representative sections obtained from euthanatized animals, representative of the four experimental groups and un-transplanted controls. The dashed lines separate brain parenchyma (*p*) from glioblastoma (*gbm*). *n* is the number of individuals of the different experimental groups, whose brains could be analyzed. (**D**) PCR profiling of DNA extracted from sections adjacent to the immune-profiled ones. Shown are amplicons representing an endogenous human gene harbored by GBM cells (*GFAP*), as well as two genes needed to make them fluorescent, *tTA* and *EGFP*, harbored by the lentiviruses employed for their engineering. (**E**) In vitro population dynamics of sister aliquots of control and Emx2-GOF U87MG preparations employed for transplantation.

**Figure 2:**
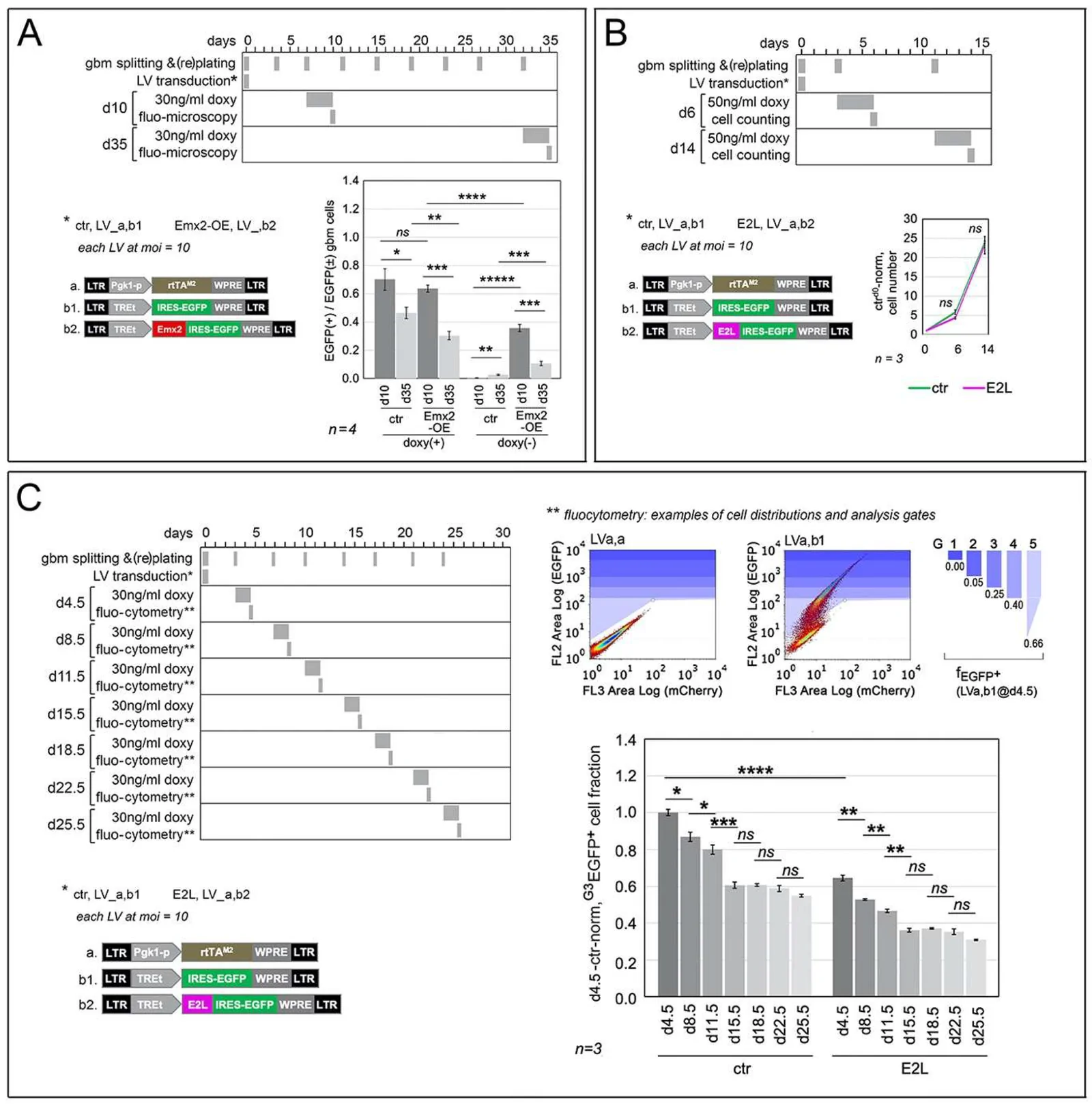
Modeling the silencing of the Emx2-encoding transgene in U87MG cells, in vitro: primary analysis. (**A**) Evaluating temporal progression of the frequency of EGFP^+^ cells, upon their transduction by the E2L-IRES-EGFP transgene or its control, delayed Tet^ON^ activation of transgenes, and final manual counting of EGFP^+^ cells. Shown are the protocol (**top**), including the lentiviruses employed (**bottom-left**), and the results (**bottom-right**). (**B**) Evaluating the impact of the doxycycline-activatable, E2L-IRES-EGFP transgene on U87MG population dynamics in vitro, with manual counting of cells. Shown are the protocol (**top**), including the lentiviruses employed (**bottom-left**), and the results (**bottom-right**). E2L, Emx2-cds-like non-coding element. (**C**) Evaluating temporal progression of the frequency of EGFP^+^ cells, upon their transduction by the E2L-IRES-EGFP transgene or its control, delayed Tet^ON^ activation of transgenes, and final cytofluorometer-assisted counting of EGFP^+^ cells. Shown are the protocol (**top**), including the lentiviruses employed (**bottom-left**) and gating criteria used for the cytofluorimetric analysis (**top/right**), as well as the results (**bottom-right**). Here (and in subsequent assays), cells were categorized as EGFP^+^, if falling in gate 3 (G3), where top 25% EGFP-expressing cells can be localized at day 4.5, upon delivery of lentiviruses a and b1 at day 0. Error bar = sem. *n* = number of biological replicates, i.e., number of independently transduced and cultured cell aliquots, originating from the same cell preparation employed for the experiment and forming each experimental group. Limited to (**A,C**), cell aliquots analyzed at different times were paired, i.e., they originated from 3 + 3 mother aliquots transduced at DIV0 with the two LV-sets in order and subsequently split, to get analyzed at different times. Statistical significance of differences between groups assessed by *t*-test (paired (**A,C**) or unpaired (**B**), homoscedastic, one-tailed). *ns*, non-significant; *, *p* < 0.05; **, *p* < 0.01; ***, *p* < 10^−3^; ****, *p* < 10^−4^; *****, *p* < 10^−5^.

**Figure 3:**
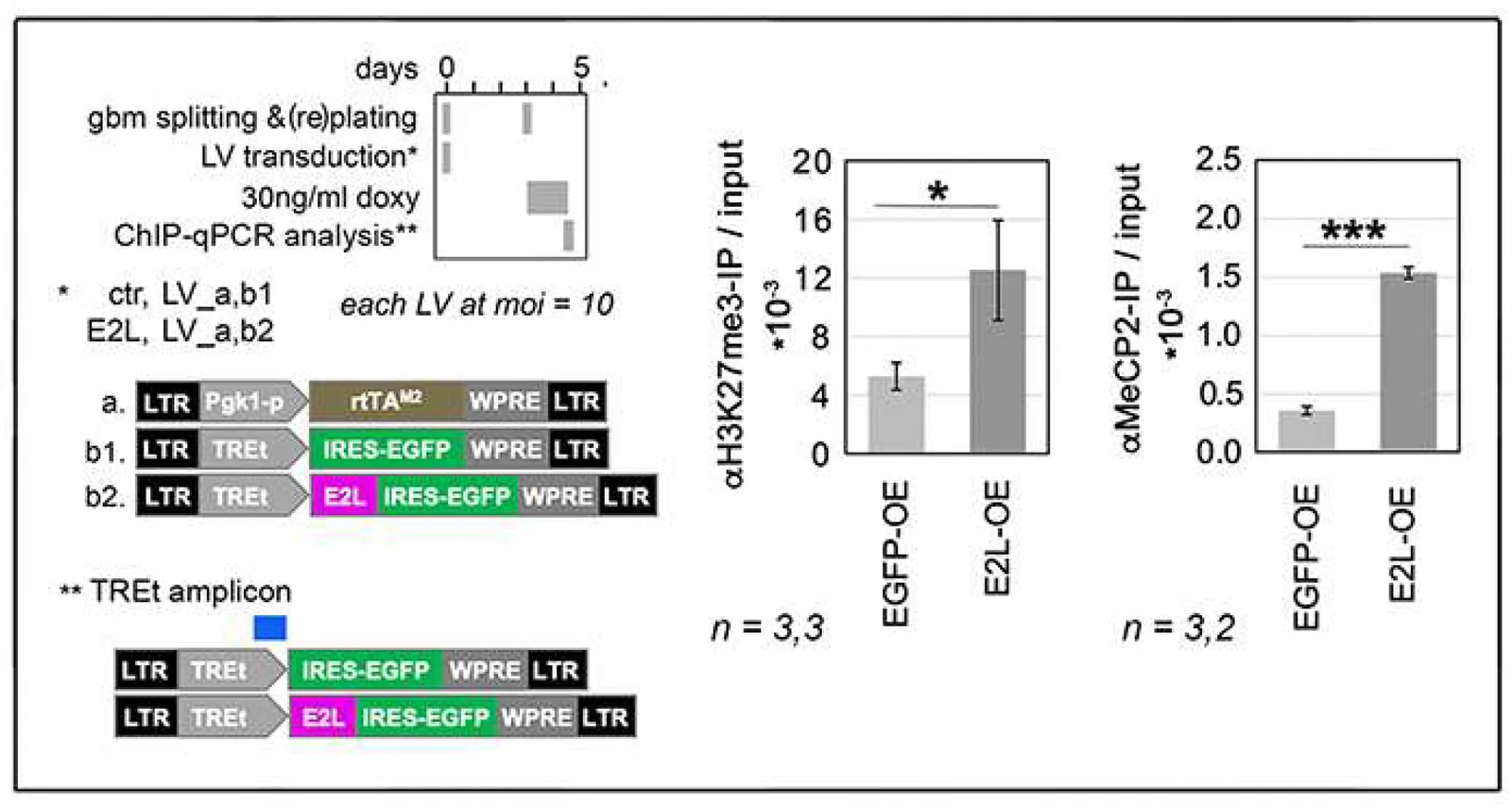
Epigenetic changes evoked by Emx2-like sequences. Absolute enrichment of the TREt amplicon for H3K27me3 and MeCP2, as assessed by chromatin-immuno-precipitation (ChIP), upon transduction of U87MG cells by TetON-controlled TREt-IRES-EGFP and TREt-E2L-IRES-EGFP transgenes and their delayed activation via doxycycline supplementation: protocol (**left**) and results (**right**). Error bar = sem. *n* = number of biological replicates, i.e., number of independently transduced and cultured cell aliquots, originating from the same cell preparation employed for the experiment and forming each experimental group. Statistical significance of differences between groups assessed by *t*-test (unpaired, homoscedastic, one-tailed). *, *p* < 0.05; ***, *p* < 10^−3^.

**Figure 4:**
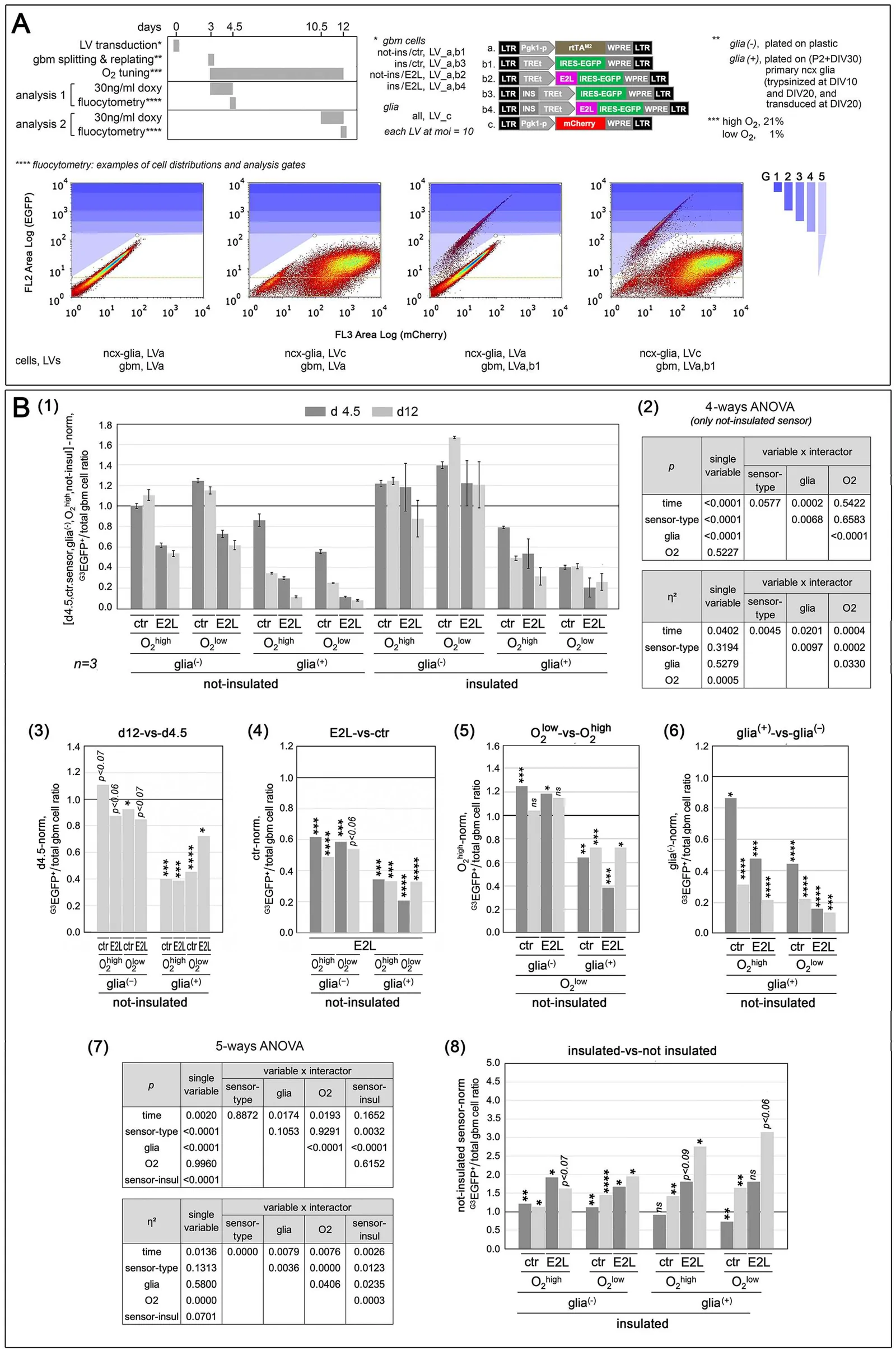
In vitro modeling of Emx2-encoding transgene silencing in U87MG cells: effects of cocultured glia, hypoxia, and transgene insulation. Evaluating temporal progression of the frequency of EGFP^+^ cells among total GBM cells, upon their lentiviral transduction (with the E2L-IRES-EGFP transgene or its control, both either naïve or 5′ insulated), their subsequent culturing (either alone or in the presence of mCherry-expressing murine pallial glia, either under normoxia or hypoxia), delayed Tet^ON^ activation of transgenes, and final cytofluorometer-assisted counting of EGFP^+^ cells. (**A**) Main protocol (**top-left**), transduction and culturing schemes (**top-right**), and examples of primary cytofluorimetric data (**bottom**). (**B**) Results. These include: (**1**) full primary results, normalized against “un-insulated/control transgene-transduced cells, cultured in the absence of glia and under normoxia, and evaluated at day 4.5”; (**2**) referring to un-insulated samples only, 4-ways ANOVA; (**3**–**6**) referring to un-insulated samples only, individual impacts of time of measure, sensor-type, presence/absence of glia and O_2_ levels on the prevalence of EGFP^+^ cells among total GBM cells, in all distinctive experimental conditions investigated; (**7**) referring to all samples, 5-ways ANOVA; (**8**) referring to all samples, impact of transgene insulation on the prevalence of EGFP^+^ cells among total GBM cells, in all distinctive experimental conditions investigated. Error bar = sem. *n* = number of biological replicates, i.e., number of independently transduced and cultured cell aliquots, originating from the same cell preparation employed for the experiment and forming each experimental group. In (**3**–**6**, **8**), statistical significance of d12-vs-d4.5, E2L-vs-ctr, O_2_^low^-vs-O_2_^high^ and glia^(+)^-vs-glia^(−)^ differences evaluated by *t*-test (unpaired, homoscedastic, one-tailed), and correction for multiple comparisons performed according to Benjamini-Hochberg (*fdr* < 0.1; *m* = 8,16 in case of (**3–6**) and (**8**), respectively). *ns*, not significant; *, *p* < 0.05; **, *p* < 0.01; ***, *p* < 10^−3^; ****, *p* < 10^−4^.

**Figure 5:**
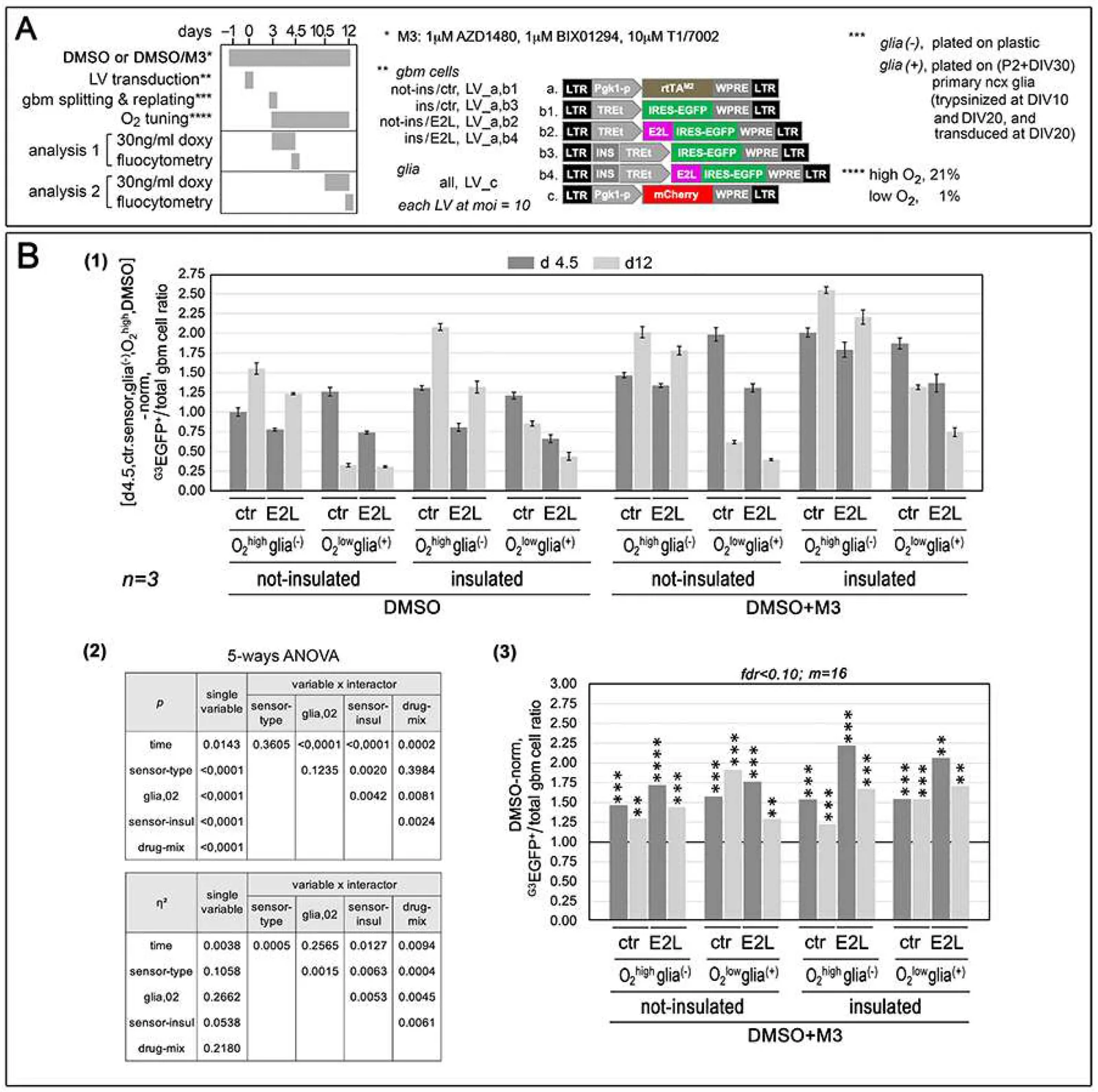
In vitro modeling of Emx2-encoding transgene silencing in U87MG cells: effects of the epigenetic drugs M3 cocktail. Evaluating temporal progression of the frequency of EGFP^+^ cells among total GBM cells, upon their lentiviral transduction (with the E2L-IRES-EGFP transgene or its control, both either naïve or 5′ insulated), their subsequent culturing (either alone/under normoxia or in the presence of mCherry-expressing murine pallial glia under hypoxia, in medium supplemented by either the DMSO vehicle or the M3 drug mix), delayed Tet^ON^ activation of transgenes, and final cytofluorometer-assisted counting of EGFP^+^ cells. (**A**) Main protocol (**left**), transduction (**mid**) and culturing schemes (**right**). (**B**) Results. These include: (**1**) full primary results, normalized against “un-insulated/control transgene-transduced cells, cultured in DMSO-supplemented medium, in the absence of glia and under normoxia, and evaluated at day 4.5”; (**2**) 5-ways ANOVA; (**3**) individual impact of the M3 drug mix on the prevalence of EGFP^+^ cells among total GBM cells, in all distinctive experimental conditions investigated. Error bar = sem. *n* = number of biological replicates, i.e., number of independently transduced and cultured cell aliquots, originating from the same cell preparation employed for the experiment and forming each experimental group. In (**3**), statistical significance of M3-vs-vehicle differences evaluated by *t*-test (unpaired, homoscedastic, one-tailed), and correction for multiple comparisons performed according to Benjamini-Hochberg (*fdr* < 0.1; *m* = 16). **, *p* < 10^−2^; ***, *p* < 10^−3^; ****, *p* < 10^−4^.

The architecture of the corresponding transgenes is shown in Figure 1, Figure 2, Figure 3, Figure 4 and Figure 5. Their full sequences are reported in Supplementary Table S1.

### 2.2. Primary Pallial Glia Cultures

Primary neocortical glial cultures were prepared from postnatal day 2 (P2) wild-type CD1 mice (Envigo, San Pietro al Natisone, Italy) housed at the SISSA Animal Facility and were used for the experiments shown in Figure 4 and Figure 5, and S2A. Pups were sacrificed by decapitation and cortices were dissected out in ice-cold 1× PBS (phosphate buffered saline) supplemented with 0.6% glucose, under sterile conditions. The meninges were carefully discarded, and each cortical hemisphere was reduced into small pieces using sharp blades, by about 10–20 cutting steps. Batches of minced neocortical tissue originating from 4 pups were individually processed under sterile conditions, as follows. After removal of excess medium by centrifugation (1 min at 200 *g*), cortical tissue was treated with DNAse I (Roche, Monza, Italy, #10104159001) in 0.05% trypsin-EDTA (ThermoFisher, Segrate, Italy, #15400054), at 37 °C for 30 min, with gentle shaking every 10 min. Then, the tissue was centrifuged for 5 min at 300 *g* and the pellet was resuspended in 10 mL of culture medium [DMEM/GlutaMAX (ThermoFisher, #10566016) + 10% heat-inactivated Fetal bovine serum (FBS) (Euroclone, Milan, Italy, #ECS180L) + 1% Penicillin/Streptomycin (ThermoFisher #15140122)], by gentle pipetting (20 to 30 times), to dissociate the suspension to single cells. Further medium was added, up to final 20 mL, and alive, dissociated cells were quantified by the Trypan blue exclusion method, using a hematocytometer. Cells were plated in T75 flasks (previously treated with 50 μg/mL poly-D-lysine (Merck, Milan, Italy, #A-003-E) for 1 h at 37 °C) at 10–15 × 10^6^ cells/flask, and incubated at 37 °C in 5% CO_2_, as described [21]. Medium was changed 2 days after cell plating, and thereafter daily, 3 times.

At day in vitro 10 (DIV10), confluent cultures were washed with 1× PBS and dissociated with 3 mL of 0.1% trypsin-EDTA (ThermoFisher #15400054), in a 5% CO_2_ atmosphere, for 10 min at 37 °C. During this time, cell detachment was monitored microscopically every 3–5 min, facilitating it by gentle tapping of the culture. Then, 5 mL of medium was added to block trypsin activity, and cells were collected by centrifugation (180 *g*, 5 min). Resuspended in fresh medium, they were plated in T75 tissue culture flasks (precoated with poly-D-lysine as above), at 2–3 × 10^6^ cells/flask, and incubated at 37 °C in 5% CO_2_. Medium was changed every 2 to 3 days.

On DIV20, confluent cells were re-trypsinized and resuspended in their culturing medium. Propedeutically to glia/glioblastoma cocultures, they were transduced with LV_Pgk1p-mCherry-wpre, at a multeplicity of infection (moi) equalling 16, sufficient to robustly label all of them, and seeded at 4 × 10^5^ cells/well, in poly-D-lysine coated six-wells plates, 2 mL medium/well. Alternatively, they were straightly plated into T75 tissue culture flasks (precoated with poly-D-lysine as above), at 2–3 × 10^6^ cells/flask.

On P30, cells were employed for glia/glioblastoma cocultures (Figure 4 and Figure 5), as detailed below, or drug toxicity assays (Figure S2A).

### 2.3. GBM Cultures

Human U87MG grade IV GBM cells were purchased from a commercial provider (Merck, Milan, Italy, #89081402) and low passage aliquots of them were employed for the experiments. In case of glia-free cultures, they were plated on Corning plasticware, generally at 20,000 cells/cm^2^ [38,000 cells/cm^2^ in case of Figure 2B, 10,000 cells/cm^2^ in case of untreated maintenance cultures], kept as adherent cultures, and trypsinized at confluency. In case of GBM/glia cocultures, GBM cells were plated on a carpet of DIV30 adhaerent glial cells, at a 1:30 GBM/glia ratio (the absolute density of glial cells was determined in parallel sister cultures, just before coculture establishment). In both monocultures and cocultures, U87MG cells were maintained in DMEM/GlutaMAX medium supplemented with 10% FBS and 1× Penicillin/Streptomycin, and kept at 37 °C, alternatively under normoxic (21% O_2_, 5% CO_2_) or hypoxic conditions (1% O_2_, 5% CO_2_), in a dedicated incubator (Eppendorf, Milan, Italy, Galaxy^®^ 14 S CO_2_, equipped with an Eppendorf #P0628-6831 oxygen sensor). When required (Figure 1E, Figure 2B and Figure S2A), numerosity of live GBM cells was assessed by the trypan blue exclusion method. At least 1000 cells per statistical replicate were counted.

### 2.4. Epigenetic Drugs

The following drugs were employed: AZD1480 (Merck #SML1505), 5-aza-2′deoxycytidine (Merck #A3656), BIX-01294 (Merck #9311), and T17002 (Merck #TA9H9804896F). They were dissolved according to Manufacturer’s instructions and administered to cells in a 1× PBS-0.12% *v*/*v* DMSO vehicle, according to Figure 5 and Figure S2 protocols.

### 2.5. GBM Cells Transplantation into Immunotolerant Mice

For this study, athymic, outbred Foxn1^nu/nu^ female mice, purchased from Envigo, and human U87GM cells were used. Seven days before surgery, cells were transduced by lentiviral vectors for Tet^OFF^-controlled overexpression of an Emx2-Ires-Egfp or an Ires-Egfp transgene and cultured in medium containing 2 mg/mL doxycyclin. 24 h before surgery, they were transferred to a doxycycline-free medium. The day of transplantation, 6-weeks-old animals were anaesthetized by intraperitoneal injection of 200 mL of saline solution, containing Ketamine (80 mg/g of body mass) and Xylazine (20 mg/g of body mass). Next, upon checking the absence of nociceptive reflexes, 5.0 μL of a solution containing 300,000 pre-engineered U87MG cells resuspended in DMEM-GlutaMAX medium, were injected through the skull into the striatum, at the stereotaxic coordinates: AP +0.5; L −1.8; V −2.8. The injection was performed by a Hamilton syringe (Hamilton, Bonaduz, Switzerland, #7105KH), lasted 20 s and was followed by slow retraction of the needle (over 10 s). Animals were left to recover in a clean cage with the help of a 37 °C thermostatic blanket and subsequently transferred to their housing cages. Limited to two out of four experimental groups, starting from four days after surgery, animals were chronically treated by temozolomide (5 mg/g body weight per day, by drinking water). Starting from 1 week after surgery, all transplanted animals were checked each day for possible appearance of symptoms of unacceptable suffering. They were euthanized upon reaching specific thresholds, i.e., when either their body mass was reduced by 20% compared to same-aged, healthy coisogenic females or they reached level 2 of the Racine’s scale [22]. Survival times were collected and employed for Kaplan-Meier statistics (in this respect, euthanasia was accounted as natural death). When feasible (i.e., in case of euthanasia), brains were dissected out and employed for subsequent immuno-histological profiling.

### 2.6. Histology and Immunofluorescence

GBM-xenografted mice brains were fixed by immersion in 4% PFA overnight at 4 °C, washed by 1× PBS 3 times, equilibrated in 30% sucrose-1× PBS solution at 4 °C, included into Killik OCT (Bio-Optica, Milan, Italy) and frozen at −80 °C. Then, they were coronally sliced at 20 μm, by a Microm (Microm, Walldorf, Germany) cryostat. Before immunofluorescence procedure, brain slices were defrosted and washed 3 times with 1× PBS in order to eliminate OCT. Next, they were incubated in blocking solution (1× PBS, 0.1% Triton X-100, 1 mg/mL BSA, 10% FBS, 0.3 M glycine) for 1 h at RT, and further incubated with 1:800 chicken polyclonal anti-GFP antibody (Abcam, Cambridge, UK, #13970) in blocking solution, overnight at 4 °C. The next day, they were washed 3 times for 5 min in 1× PBS-0.1% Triton X-100 and incubated for 2 h at RT with 1:800 488 Alexa Fluor^TM^-conjugated goat anti-chicken antibody (Invitrogen, Segrate, Italy, #A-11039) and 1 mg/mL DAPI in blocking solution. After three additional 5 min washes in 1× PBS-0.1% Triton X-100, sections were mounted with Vectashield^®^ Antifade Mounting Medium (Vector Laboratories, Newark, CA, USA, #H-1000). Immunofluorescences were photographed on a Nikon Eclipse Ti confocal microscope equipped with a 20× air objective and a Hamamatsu camera. Images were collected by NIS-Elements 5.42 software (Nikon Europe, Amstelveen, the Netherlands) as .nd2 files and processed using Photoshop CS2 (Adobe, San Josè, CA, USA) software.

### 2.7. Genomic DNA Extraction and Its PCR Analysis

Genomic DNA was extracted from brain slices or U87MG cells by the FlexiGene DNA Kit (Qiagen, Hilden, Germany) according to Manufacturer’s instructions, resuspended in water, and quantified by a DS-11 spectrophotometer (DeNovix, Wilmington, DE, USA). Genomic DNA derived from brain sections was analyzed by end-point PCR, 60 ng/reaction, by means of GoTaq^(R)^G2FlexiDNA polymerase (Promega, Milan, Italy), according to Manufacturer’s instructions. U87MG DNA was profiled by qPCR, 60 ng/reaction, in technical triplicate, by means of the SsoAdvanced SYBR Green SupermixT platform (Biorad, Segrate, Italy), according to Manufacturer’s instructions. Lentiviral integration rates were determined as ratios between lentiviral TREt and genomic OTX2 amplicons. Statistical significance assessed by *t*-test (one-tailed, unpaired). Primer sequences are detailed in Supplementary Table S2.

### 2.8. Quantification of EGFP^+^ Cells: Manual

U87MG cells, pre-transduced with Tet^ON^ activatable, EGFP-encoding transgenes, were challenged by a mild and short doxycycline stimulation (see Figure 2A protocol for details) and eventually profiled by epifluorescent microscopy. For this purpose, they were dissociated by trypsinization, allowed to acutely re-attach to the bottom of a petri dish (pre-treated with 250 μg/mL poly-D-lysine (Merck #A-003-E) for 1 h at 37 °C) for 1–2 h, and profiled for EGFP expression, by direct fluorescence and dark/bright-field microscopy. Fluorescent cells were photographed on a Nikon Eclipse TI confocal microscope (Nikon Europe, Amstelveen, The Netherlands) equipped with a 20× air objective and Hamamatsu camera (Hamamatsu Photonics, Arese, Italy). Pictures were acquired as .nd2 files and analyzed by NIS-Elements 5.42 software, by an operator blind to sample identity. For each dissociated cell culture sample, about 1000 cells collected from at least 10 randomly assorted photographic fields were scored. Frequencies of EGFP^+^ cells were averaged and sem’s were calculated. Results were normalized and statistically analyzed as detailed in Legend to Figure 2.

### 2.9. Quantification of EGFP^+^ Cells: Cytofluorimetric

U87MG cells, pre-transduced with Tet^ON^ activatable, EGFP-encoding transgenes and mixed–where appropriate–with mCherry-expressing glial cells, were challenged by a mild and short doxycycline stimulation (see Figure 2, Figure 4, Figure 5 and Figure S2 protocols for details) and eventually profiled by cytofluorometry. For this purpose, they were washed by 1× PBS, trypsinized, spun at 200 *g*, and resuspended at 1 × 10^6^ cells/mL, in a dedicated flow cytometry buffer (a phenol red-free medium including 1× PBS, 25 mM HEPES, and 2% FBS). Cell suspensions were filtered by 70 μm filters (pluriSelect, Leipzig, Germany, #43-10070-70), transferred to flow cytometer tubes (pluriSelect #05-03040-01), and profiled by a Biorad S3 Cell cytofluorimeter. First, forward scatter (FSC) and side scatter (SSC) parameters were used to exclude debris and cell aggregates. Next, to categorize GBM cells as EGFP^+^ or EGFP^−^, analytical gates G1–G5 were set with reference to distinct EGFP fluorescence intensity thresholds, to include the top 1%, 5%, 25%, 40% and 66% EGFP^+^ cells of the “d4.5 control GBM preparations” mentioned in Figure 2C (as envisaged, these gates did not include mCherry^+^ cells of Figure 4 and Figure 5). Because of its selectivity against autofluorescence and sensitivity to experimental perturbations subject of investigation, G3 was selected and hereafter employed for all GBM cells categorizations. The resulting ^G3^EGFP^+^ cell fractions were quantified as readouts of transgene inducibility. Primary data analysis was performed by Prosort 1.6 software (Biorad). Assays were generally run at least in biological triplicate and 100,000 cells per sample were scored. Prevalences of ^G3^EGFP^+^ cells among GBM cells were averaged and sem.s were calculated. Results were normalized and statistically analyzed as detailed in Legends to Figures.

### 2.10. Chromatin ImmunoPrecipitation (ChIP) qPCR

Chromatin Immunoprecipitation-quantitative Polymerase Chain Reaction assays (ChIP-qPCRs) were performed for H3K27me3 and MeCP2 by the MAGnifyTM Chromatin Immunoprecipitation System kit (Invitrogen #49-2024) and according to Manufacturer’s instructions. Chromatin aliquots were prepared each from 3.5 × 10^6^ U87MG cells. Following trypsinization, the cell suspension was transferred to a centrifuge tube and spun at 200 *g* for 5 min. The supernatant was discarded and the pellet was resuspended in 500 μL of 1× PBS. Cells were fixed with 1% formaldehyde for 10 min at RT and the reaction was quenched by 1.25 M glycine for further 5 min at RT. Fixed cells were washed 3 times in ice-cold PBS and resuspended in 170 μL in Lysis Buffer plus protease inhibitors, by gentle vortexing. The suspension was incubated on ice for 10 min. Aliquots of crosslinked chromatin originating from 3.5 × 10^6^ cells were sheared to 300–500 bp fragments, using a Soniprep 150 apparatus (Medical and Scientific Structures, MSE, Hearthfield, United Kingdom) set as follows: on ice; 5 s ON, 55 s OFF; oscillation amplitude 7 μm; 3 cycles. The quality of sonicated chromatin was checked by agarose gel electrophoresis, after 100 mg/mL RNAse treatment at 37 °C overnight and 1 mg/mL proteinase K incubation at 55 °C for 1 h.

Next, αH3K27me3 (rabbit polyclonal, Merck #07-449) and αMecP2 (rat polyclonal IgG2a serotype, Active Motif, Carlsbad, CA, USA, #61291) antibodies, both 10 μg/reaction, were incubated with Protein A/G Dynabeads^®^ (ThermoFisher) in Diluition Buffer, in a final volume of 100 μL, for 2 h at 4 °C, keeping the tubes in a rotating device. Immediately after, for each ChIP reaction, one subaliquot of sonicated chromatin originating from 8 × 10^4^ cells was incubated with 10 μg of Dynabeads-bound antibody at 4 °C overnight. Bead-bound immunocomplexes were precipitated by a magnet, washed three times with ice- cold wash IP buffer 1 and twice with ice-cold wash IP buffer 2, 5 min per wash on a rotating device, and resuspended in reverse cross-linking buffer. The resulting suspension was incubated at 55 °C for 15 min and treated with a magnet. Supernatant DNA was finally transferred to a fresh tube and purified by further magneto-precipitation. Chromatin was eluted by Elution buffer at 65 °C for 20 min.

Immunoprecipitations were performed in biological triplicates, by using the same chromatin lysate for αH3K27me3 and αMeCP2 immunoprecipitation/sample. Sister preparations of not-immunoprecipitated, crosslinked chromatin (input chromatin) were similarly processed and employed for standard normalization of results. 1/5 of each immunoprecipitated (IP) DNA sample was scored by qPCR, for its TREt amplicon content, in technical triplicate. Results were evaluated by *t*-test, as detailed in Legend to Figure 3.

### 2.11. Statistical Evaluation of Results

Full details of it are provided in Legends to Figures.

## 3. Results

### 3.1. Emx2 Overexpression Mitigates the Outcome of Orthotopic GBM Xenograft to Immunodeficient Mice, While Not Eradicating the Tumor

To ascertain the anti-glioblastoma Emx2 therapeutic power in vivo, we compared survival rates of immunocompromised mice alternatively xenografted by Emx2-OE or control GBM cells. For this purpose, we engineered U87MG GBM cells by lentiviral vectors and Tet^OFF^ technology, making them to conditionally overexpress a dicistronic transgene encoding for Emx2 and an EGFP reporter, or a control, EGFP-encoding transgene. After lentiviral transduction, we kept the genes silent for one week, by doxycycline medium supplementation. One day before transplantation, we removed the antibiotic, to activate the transgenes. We transplanted engineered cells into the striatum of juvenile immunodeficient mice, 300,000 ones per animal. Four days after transplantation, we administered temozolomide (TMZ) orally to 27 out of 54 transplanted mice; in this way we created four groups: the “control group” (*n* = 14), the “Emx2-OE group” (*n* = 13), and the corresponding groups also treated with TMZ (*n* = 14, 13, respectively). We systematically scored mice for distress and suffering symptoms and, when needed, we humanely sacrificed them, according to standard procedures. We annotated survival times of transplanted mice over almost four months, equaling euthanasia to natural death (Figure 1A). We found that mice transplanted with Emx2-OE GBM cells displayed a median survival time of 56 days, compared to 35 days of the control group (*p* < 0.0001, *n* = 14, 14). Emx2 overexpression also outperformed temozolomide treatment, which increased such median survival time up to only 45 days (*p* < 0.0001, *n* = 14, 13). Moreover, no statistically significant difference was detectable between “Emx2-OE” mice having received TMZ or not (*p* > 0.05, *n* = 14, 13) (Figure 1B). To note, 13/14 mice of the Emx2-OE group overcome the maximum survival time of ctr mice, 46 days, two of them exceeding 100 days. In a similar way, 10/13 mice of the Emx2-OE/TMZ group overcome the maximum survival time of TMZ-treated mice, 54 days, two of them exceeding 100 days. Taken together, these results indicate that Emx2 exerted a pronounced antiblastic activity in vivo, even stronger than the TMZ treatment. However, all animals transplanted by Emx2-OE cells died less than 120 days after GBM transplantation, meaning that Emx2 robustly mitigated the aggressiveness of the tumor, while—however—not eradicating it.

We reasoned that the limited power displayed by our therapeutic transgene in vivo could originate from its silencing or GBM desensitization to the Emx2 protein. To cast light on this point, we sliced all available brains of xenografted mice (harvested from euthanized animals), and we analyzed the distribution of anti-EGFP immunoreactivity as a proxy of transgene expression. We found that, regardless of TMZ treatment, a fraction of tumor cells pre-engineered by control lentivirus still expressed EGFP, while none of their Emx2-OE counterparts did (Figure 1C). Notably, when interrogated by PCR, genomic DNA extracted from slices adjacent to the immunoprofiled ones was robustly positive for markers of both control and Emx2-encoding lentiviruses, respectively (Figure 1D). Remarkably, if kept in vitro, sister aliquots of Emx2-OE cells employed for xenografting collapsed within only 2 weeks, differently from exponentially expanding controls (Figure 1E). All this suggests that: (a) all GBM cells engineered for Tet^OFF^-controlled Emx2 over-expression had received the two intended lentiviruses, resulting in a reduction of the tumor burden weighting on transplanted mice, (b) however a substantial fraction of them had undergone pronounced transgene silencing, specifically when transferred to brain parenchyma.

### 3.2. Primary Modeling of Emx2 Transgene Silencing In Vitro

To model mechanisms inducing transgene silencing and develop devices suitable to counteract them, we set up a dedicated in vitro approach. We transduced GBM cells with the Tet^ON^-controlled therapeutic transgene or its control counterpart, both associated to an IRES-EGFP reporter, and we kept them inactive for a prolonged time (up to 35 days). This approach was based on the assumption that extended transgene inactivity would facilitate the onset of silencing, a phenomenon that had not been detected during shorter in vitro culture periods [17]. Next, we interrogated these cultures for their ability to activate the transgene, at day 10 and 35 after lentiviral transduction, by visually scoring their EGFP-expressing cell fraction upon delayed medium supplementation with doxycycline. This antibiotic was administered at low concentration for a short time, to limit the cytocide/cytostatic effect elicited by Emx2 and make the EGFP-expressing cell fraction a reliable proxy of transgene transcription. As expected, inducibility progressively declined over time for both transgenes, with a substantially greater reduction observed for the Emx2 construct (Figure 2A). This suggests that the architecture itself shared by our vectors predisposes the transgenes to silencing, while Emx2 sequences exacerbate this phenomenon. Moreover, some EGFP accumulation above the detectability threshold took also place in preparations kept in a doxy-free medium, likely reflecting leakiness of the Tet^ON^ machinery. Despite the increasing trend, the EGFP^+^ cell fraction remained negligible in control-transduced cultures, even on day 35 (d35). Conversely, it was quite high in Emx2-transgene-transduced preparations at d10, and subsequently declined, likely reflecting an initial cytostatic and then cytotoxic effect elicited by Emx2 (Figure 2A). Because these effects could confound the interpretation of changes in the EGFP-positive fraction, we generated a modified Emx2-IRES-EGFP construct in which subtle mutations abolished Emx2 protein translation, thereby eliminating its effects on cell proliferation and survival, while preserving the CpG-rich sequences located downstream of the Emx2 transcription start site (TSS), which were hypothesized to promote silencing (Table S1; Figure 2B). With this new tool, E2L-IRES-EGFP, (and its EGFP control) GBM cells were interrogated. To increase the discriminative power of the assay, the sensor transgene was challenged by a milder and shorter doxycycline supplementation. To score larger samples and better standardize their categorization, cells were profiled by automated cytofluorometry. Finally, to maximize the sensitivity of the assay to ongoing gene silencing, cells were classified as EGFP^+^ only if falling into a specific G3 gate, corresponding to the top 25% fluorescent cells of the youngest control samples. GBM cells were profiled over a large time window, spanning from 4.5 up to 22.5 days after lentiviral transduction. Remarkably, all lentiviral constructs underwent progressive silencing, which reached the plateau 15 days after lentiviral transduction. Throughout the entire observation period, constructs containing Emx2-derived sequences consistently exhibited lower inducibility than their corresponding controls (Figure 2C).

We suspected that selective inactivation of the Emx2 transgene might have been triggered by specific sequences associated to such transgene, including a large subset of the CpG island that spans I exon of the natural gene and its surroundings. Results of aH3K27me3- and aMeCP2-ChIP assays suggested that transgene silencing was likely achieved via redundant mechanisms, mediated by both H3K27me3 and MeCP2 marks, exacerbated in case of Emx2-like sequences (Figure 3). This scenario is consistent with Mortimer et al. [18], reporting that distinct glioblastoma tumors silence the endogenous Emx2 gene via both trimethylation of H3K27 and C-methylation of its DNA [23,24,25].

### 3.3. Assessing Hypoxia and Glia Contribution to Emx2 Transgene Silencing In Vitro

We hypothesized that two key factors can substantially enhance silencing of our therapeutic transgene in xenografted mice: (a) the low O_2_ levels characterizing the tumor niche, and (b) the interaction between tumor and surrounding glia. The former can deplete the available aketo-glutarate pool [26], jeopardize the activity of Tet1,2,3 ^5meC^DNA-oxydases and KDM6B-H3K27me_2,3_ and KDM4E-H3K9me_2,3_ demethylases (which require aketo-glutarate as an obliged cofactor [27]) and result in un uprise of the corresponding silencing marks, ^5meC^DNA, H3K27me_3_, and H3K9me_3_ (Figure S1A). The latter may lead to a strong upregulation of the Jak2/Stat3 pathway, originating from mutual, glia/GBM paracrine stimulation via IL6-like cytokines, and result in a self-sustaining uprise of EZH2-H3K27me0,1,2-methyltransferase, a decline of KDM6B-416 H3K27me1,2 demethylase, and-therefore-a further pronounced upregulation of H3K27me3 (see Figure S1B and references therein for details).

To test this hypothesis, we cultured GBM cells harboring the Tet^ON^-controlled TRE-E2L-IRES-EGFP sensor (E2L) or its TRE-IRES-EGFP control (ctr), either with aged (P2+DIV30) pallial glial cells constitutively expressing an mCherry tracer (glia^(+)^), or kept alone (glia^(−)^), in the presence of 21% O_2_ (O_2_^high^) or 1% O_2_ (O_2_^low^). Then, 4.5 and 12 days later, we evaluated the fraction of (mCherry^−^) GBM cells able to achieve sustained sensor activation (i.e., falling into the G3 cytofluorimetric gate), following delayed and mild doxycycline stimulation (Figure 4A).

Multivariate analysis of results (Figure 4B (1), left half) showed that this fraction was primarily sensitive to time of measure, sensor-type and glia, but—apparently—not to O_2_ levels (Figure 4B (2)). Mono-variate analysis of results allowed to further detail the emerging scenario, as follows. Specifically in the presence of glia, d4.5 values largely exceeded the corresponding d12 ones (Figure 4B (3)). E2L values were always smaller than the corresponding ctr values and this phenomenon was particularly prominent in the presence of glia (Figure 4B (4)). Lower O_2_ levels reduced the ^G3^EGFP^+^/total GBM cell fraction, however only in the presence of glia, while not impacting (or moderately increasing) it in pure GBM cultures (Figure 4B (5)). The presence of cocultured glia always reduced the ^G3^EGFP^+^/total GBM cell fraction, and this reduction was more pronounced in case of E2L sensors, at later times, as well as under low oxygen (Figure 4B (6)). Of note, the statistical interaction among (a) glia on one side and (b) time of measure or O_2_ levels on the other was formally significant (Figure 4B (2)). All this translated into a dramatic activity decrease which characterized the E2L-sensor, while moving from a normoxic/glia-free environment to a hypoxic/glia-rich one (by 5.4- and 5.5-folds, at d4.5 and d12, respectively with *p* < 1.7 × 10^−5^ and *p* < 5.7 × 10^−5^). A similar, albeit delayed and less dramatic decline also characterized the ctr-sensor (by 1.8- and 4.4-folds, at d4.5 and d12, respectively, with *p* < 4.9 × 10^−5^ and *p* < 4.4 × 10^−5^) (Figure 4B (1)). In this respect, it is reasonable to hypothesize that (a) ctr-sensor inhibition is due to elements shared by the two sensors and/or harbored by their Pgk1-rtTA^M2^ driver, (b) supplementary inhibition of the E2L sensor reflects cis-silencing activity of Emx2 sequences. Any devices able to antagonize these two effects in vitro might be of valuable help to enhance Emx2 therapeutic power in vivo.

### 3.4. Mitigating Glia- and Low Oxygen-Dependent Silencing of Lentivirus-Encoded Emx2-Like Sensor Transgene by Insulation

To counteract the negative impact exerted by co-cultured glia and low O_2_ on delayed activatability of our sensor transgenes, we firstly employed a miniaturized, 0.7 kb long Ubiquitous Chromatin Opening Element (UCOE), taken from the human A2B1/CBX3 intergenic region [28]. We placed it upstream of the TREt promoter of our sensor transgenes, in order to keep them in an “open chromatin” configuration, by recruiting RNA polymerase II and promoting basal transcriptional read-through, while preserving the inducibility of the TREt promoter [28]. Next, we compared performances of the resulting “insulated” sensors with their not-insulated ancestors, again at d4.5 and d12, in GBM cells cultured alone or in the presence of glia, under high or low oxygen (Figure 4A).

Multivariate analysis of results (Figure 4B (1), full graph) substantially confirmed the primary impact of time, sensor-type and glia variables on sensor activatability, as well as their statistical interaction pattern, as already documented in un-insulated cultures (compare Figure 4B (2) and (7)). Direct comparison between insulated and non-insulated sensors further demonstrated an overall beneficial effect of UCOE insulation on transgene inducibility. This improvement was particularly evident for the E2L sensor, especially in the presence of glia and at later time points (Figure 4B (8)), consistent with the significant interactions observed between insulation and sensor type, as well as between insulation and glia (Figure 4B (5)). Remarkable were the insulation-driven gains in the ^G3^EGFP^+^/total GBM cell ratio, displayed by the E2L-type sensor at d12.0, (a) under low oxygen and no glia (1.95 ± 0.36, with *p* < 0.03, *n* = 3, 3), (b) under high O_2_ and in the presence of glia (2.76 ± 0.74, with *p* < 0.04, *n* = 3, 3), as well as (c) upon combined hypoxic/coculturing treatment (3.15 ± 1.00, with *p* < 0.06, *n* = 3, 3).

### 3.5. Improving Performances of Lentivirus-Encoded Emx2-Like Sensor Transgene in the Presence of Glia- and Under Low Oxygen, by Pharmacological GBM Cells Treatment

We further tried to mitigate the scarce, delayed responsivity displayed by ctr- and E2L-EGFP sensors by means of a cocktail of three FDA-approved drugs, namely T1/7002 (an EZH2-H3K27me_0,1,2_ methyltransferase inhibitor lowering H3K27me3 levels), BIX-01294 (a G9a-H3K9MT inhibitor lowering H3K9me3 levels), and AZD-1480 (a Jak2 inhibitor dampening pStat3 signaling), supposed to antagonize mechanisms triggered by co-cultured glia and hypoxia and leading to chromatin silencing in GBM cells (Figure S1).

First, a mix including these three drugs, named M3, was set, selecting a concentration vector which preserved glia and GBM cells vitality under hypoxia, while allowing for proper functioning of the control sensor (see Supplementary Results and Figure S2).

Next, to evaluate the M3 impact on the EGFP sensor expression in different operational conditions, GBM cells were transduced by ctr- or E2L-type sensors, naïve or insulated, and alternatively (a) cultured alone under high oxygen, or (b) co-cultured with glia in hypoxic conditions. Moreover, they were alternatively exposed to the M3 mix or its DMSO vehicle, immediately before LV transduction and until cytofluorometric profiling, at d4.5 and d12 (Figure 5A).

Five-ways ANOVA analysis of results showed that global M3 impact was significant (*p* < 0.0001) and prominent (h^2^ = 0.22). Moreover, M3 interacted significantly with time (*p* < 0.0002), glia/O2 (*p* < 0.0081) and insulation state of the sensor (*p* < 0.0024) (Figure 5B (1) and (2)). Specifically, for each combination of the other four independent variables, the M3 mix elicited a statistically significant (from *p* < 0.01 to *p* < 0.0001) increase of the ratio between ^G3^EGFP^+^ and total GBM cells (from ≈1.25× to ≈2.25×). Remarkably, this increase was conspicuous in case of d12 cells transduced with the insulated E2L-sensor and cocultured with glia under hypoxia (>1.7×, with *p* < 0.01) (Figure 5B (5)). Despite missing statistical interaction between M3 and sensor-type, this suggests that, associated to transgene insulation, M3 can prevent the progressive decline of E2L-sensor responsivity evoked by a hypoxic/glia-rich environment. Actually, the normalized ^G3^EGFP^+^/total GBM cell ratio in d12 hypoxic/glia-rich, “insulated”/M3-treated cultures, 0.74 ± 0.06, almost equaled the same ratio in d4.5 normoxic/glia-free, “un-insulated”/DMSO treated ones, 0.78 ± 0.02 (Figure 5B (1)).

## 4. Discussion and Conclusions

In this study, we found that overexpressing an *Emx2* transgene in U87MG glioblastoma cells transplanted into the brain of immunotolerant mice increased the survival time of these animals, exceeding the therapeutic benefit achieved with temozolomide (Figure 1). However, this treatment was not sufficient to eradicate the tumor, apparently because of transgene silencing in vivo (Figure 1). We also found that the *Emx2* transgene and a dedicated sensor of its transcriptional competence underwent progressive silencing during prolonged in vitro GBM culture (Figure 2 and Figure 3), a process that was further exacerbated by coculture with murine pallial glia under hypoxic conditions (Figure 4). Finally, we showed that transgene hemi-insulation by an UCOE element as well as the use of an ad hoc set-up mix of epigenetic drugs largely fixed the activatability decline undergone by the *Emx2*-expression sensor in aging GBM cells (Figure 5).

The increase in the survival time of xenografted animals we achieved by *Emx2* overexpression was conspicuous (56 vs. 35 days), outperforming temozolomide, which was administered here at doubled dosage compared to standard human therapy [29]. However, mice did not survive, they died by <120 days after tumor transplantation. This is apparently at odd with Mortimer et al. [18], according to whom a moderate activation of *Emx2* in DBTRG-05MG GBM cells sufficed to inhibit their in vivo expansion over >40 days. This discrepancy might be due to differential sensitivity to Emx2 peculiar to U87MG and DBTRG-05MG lines. It might further reflect a stronger Emx2 expression elicited in DBTRG-05MG cells. Differently from U87MG cells, in fact, these cells were preselected before transplantation, filtering out those expressing little or no transgene products [18].

In principle, the long-term persistence of deadly tumor cells in brains of our Emx2-OE-GBM transplanted animals might originate from selective expansion of rare GBM cells (1) not transduced by the therapeutic viruses or (2) affected by delayed loss-of-function mutations in the therapeutic transgene. However, the collapse of sister aliquots of xeno-transplanted, Emx2-GOF cell preparations alternatively kept in vitro, as well as the in vivo distribution profile of DNA and expression products of ctr- and *Emx2*-transgenes (Figure 1C,D) convinced us that *Emx2* inability to eradicate the tumor could mainly originate from its specific in vivo silencing.

Modeling these phenomena in vitro gave results consistent with this hypothesis. In fact, compared to controls, *Emx2* sequences turned out to be intrinsically more prone to acquire suppressive epigenetic marks (Figure 3) and limit the expression of a transgenic reporter containing them (Figure 2C and Figure 4B (4)). Coculture with glial cells consistently exacerbated this effect (Figure 4B (6)), whereas reduced oxygen tension, comparable to that reported for hypoxic glioblastoma niches [30], was effective only in the presence of glia (Figure 4B (4)). To note, weakness of E2L-Ires-EGFP-driven fluorescence could originate not only from poorer transcription. In fact, albeit Ires-EGFP- and E2L-Ires-EGFP are both translated in the same IRES-dependent fashion, steric hindrance by the E2L-5′UTR might selectively dampen translation of E2L-Ires-EGFP-mRNA compared to its Ires-EGFP control (Figure 2C, Figure 4B (1), and Figure 5B (1)). On the other side, with regard to the negative impact that hypoxia can exert on synthesis and maturation of EGFP [31], as these phenomena specifically occur at <0.06% O_2_ [31], i.e., well below our 1–20% working interval, that should not be a matter of concern.

However, *Emx2* sequences are only a part of the problem, as even ctr-sensor sequences (*shared with the E2L-sensor*) evoked a progressive decline of sensors responsivity (Figure 2C), which was made more and more pronounced by glia (Figure 4B (3)), and—interestingly—combined glia/hypoxia (Figure 4B (6)).

Remarkably, although not abolishing progressive gene silencing, both UCOE protection and the use of the M3 drug mix ameliorated the performances of both sensors, with insulation especially effective in enhancing E2L-Ires-EGFP signals (Figure 4B (8) and Figure 5B (3)). All that encourages us to pursue the approach implemented in the present study, of course with specific improvements. For example, we expect that a better insulation strategy, including the placement of an additional insulator on the 3′ side of the EGFP transgenes as well as the bilateral insulation of the accessory Pgk1-rtTA^M2^-WPRE transgene in charge of their transactivation [employed as “not insulated” throughout this study (Figure 4A)] should be definitively beneficial. On the other side, we envisage that the implementation of our insulation/epigenetic strategy in vivo should let further synergic advantages emerge, not appreciable while focusing on simple prevention of transgene silencing in vitro. For example, that is the case of Jak/Stat inhibition, in the light of Stat3 ability to impair anti-tumor immunity (reviewed in [32]) and promote tumor malignancy [33,34,35,36,37].

Finally, we want to stress that this is just a proof-of-concept study, run within an intentionally simplified operational framework, consisting in lentiviral delivery of a constitutively expressed *Emx2* transgene to GBM cells prior to transplantation. Two its main limits. First, Emx2 overexpression in glutamatergic neocortical neurons (as resulting from real in vivo delivery of a constitutive promoter-driven therapeutic transgene) would be hardly acceptable, as highly toxic [17]. Second, albeit a powerful tool for flexible genetic engineering of somatic cells, recombinant lentiviruses would be a poor choice for in vivo engineering of GBM, because of their intrinsic genotoxicity and inability to tackle all infiltrating tumor cells. Luckily, both limits can be overcome. In fact, driven by a promoter selectively active in tumor stem cells, *Emx2* retains its capability to suppress GBM in vitro [17]. Moreover, lentiviral vectors can be successfully replaced by highly neurotropic, herpetoviral vectors, selectively active in intermitotic cells (Zucco and Mallamaci, unpublished results). The integrated exploitation of these devices (and further refined epigenetic-targeting strategies) will be addressed in future follow-up studies.

## Data Availability

All primary data related to this study are reported in Supplementary Materials.

## Supplementary Materials

The following supporting information can be downloaded at: https://www.mdpi.com/article/10.3390/biomedicines14092010/s1: Supplementary Results: Setting-up a drug-mix suitable to prevent silencing of lentivirus-encoded sensor transgenes in GBM cells [38,39,40,41,42,43]; Supplementary Figure S1: Putative mechanisms leading to gene silencing in hypoxic GBM/glia cocultures. (**A**) Impact of hypoxia on effectors controlling the epigenetic state of DNA and H3 histones [18,44,45]. (**B**) Autocrine and crossed/paracrine activation of Jak2/Stat3 signaling in GBM/glial cocultures and its impact on effectors controlling H3K27 methylation state [46,47,48,49,50,51,52]; Supplementary Figure S2: Setting-up an epigenetic drug mix suitable to antagonize silencing of the therapeutic *Emx2* transgene. (**A**) Impact of AZD-1480, aza-deoxyC, BIX-01294 and T1/7002, alone or combined, on population dynamics of primary cultures of murine neocortical glia and U87MG GBM cells, grown over 6 days under hypoxia: protocol (left) and results (right). (**B**) Impact of distinctive mixes of the four drugs listed above on the prevalence of EGFP+ cells among U87MG cells transduced by a Tet^ON^-controlled, lentiviral TREt-IRES-EGFP transgene: protocol (top) and results (bottom). Shown are: (1) and (3), frequencies of EGFP+ cells, and (2) average proviral copies per cells, all detected upon delivery of each lentivirus at moi = 10 and cells exposure to distinctive drug mixes over 3d–4.5d. Error bar = sem. n = number of biological replicates, i.e., number of independently transduced and cultured cell aliquots, originating from the same cell preparation employed for the experiment and forming each experimental group. Statistical significance of differences between groups (drug-vs-vehicle in graphs (1–4) of (A)), assessed by ttest (unpaired, homoscedastic, two-tailed). ns, non-significant; ∗, *p* < 0.05; ∗∗, *p* < 10^−2^; ∗∗∗, *p* < 10^−3^; ∗∗∗∗∗, *p* < 10^−5^.; Supplementary Figure S3: Uncropped Agarose Gels Referred to in Figure 1D; Supplementary Table S1: Sequences of LV cargo Vectors; Supplementary Table S2: Sequences of PCR Primers; Supplementary Table S3: Full primary data.
